# Supplementary material for: Prediction of the functional impact of missense variants in BRCA1 and BRCA2 with BRCA-ML
Source: NPJ Breast Cancer. 2020 Apr 29;6:13. doi: 10.1038/s41523-020-0159-x (PMC7190647; doi:10.1038/s41523-020-0159-x)
Supplement: Supplementary file 1 — Supplemental Data Set 1 Description. [file 41523_2020_159_MOESM1_ESM.pdf]

Supplemental Data Set 1 Description.

This table contains BRCA-ML scores for all possible missense mutations that can occur by single nucleotide variation in the BRCA1 and BRCA2 Genes. It also contains all the scores from the individual missense prediction algorithms that were used as inputs to derive the BRCA-ML score.

Supplemental Data Set 2 Description.

Homologous recombination deficiency repair scores for novel BRCA2 missense mutations.
